# Beyond Simple Averaging: Combining Scores, Reliabilities, and Validities of Multiple Intelligence Tests

**DOI:** 10.3390/jintelligence14070122

**Published:** 2026-07-01

**Authors:** Dominik Weber, Nicolas Becker, Frank M. Spinath, Marco Koch

**Affiliations:** Department of Individual Differences & Psychodiagnostics, Saarland University, 66123 Saarbrücken, Germany; nicolas.becker@mx.uni-saarland.de (N.B.); f.spinath@mx.uni-saarland.de (F.M.S.); marco.koch@uni-saarland.de (M.K.)

**Keywords:** intelligence assessment, IQ, composite, reliability, validity, simulation study

## Abstract

In applied diagnostics, scores from multiple intelligence tests are often combined by simple arithmetic averaging. Although convenient, this practice is statistically imprecise: it neglects (a) that tests are positively correlated but not identical, each capturing somewhat different aspects of intelligence, and (b) that combining correlated measures reduces variance. Consequently, arithmetic means underestimate intelligence above the IQ scale center and overestimate it below the center. Such distortions can lead to serious misjudgments in high-stakes assessment, such as in educational placement or forensic evaluations of legal culpability. To obtain exploratory evidence on the prevalence of simple averaging practice, we surveyed *n* = 75 psychologically educated individuals familiar with IQ metrics. In response to a case vignette requiring the combination of several IQ scores, 58 participants (77.33%) applied simple averaging, and none considered intercorrelations and variance reduction. Therefore, the aim of this article was to propose the application of a more valid method for combining scores (as well as corresponding reliabilities and validities) of multiple tests. To validate the method, we performed Monte Carlo simulations, covering a wide range of test characteristics. Results showed virtually perfect accuracy (*r* = 1.00, *p* < .001), and the outcomes were robust against variations in the number of tests to be combined, score distributions, and intercorrelations. To facilitate adoption in practical diagnostics, we developed and introduced an open-source R package and an accompanying open-access Shiny web application.

## 1. Introduction

In applied settings, scores, reliabilities, and validities of multiple intelligence tests are frequently combined by means of simple averaging. Although convenient, this practice is statistically imprecise. The present article proposes a practical implementation of a method for combining test values based on established statistical principles and introduces an accompanying R package, along with a Shiny web application, that facilitates its practical application. The method was validated by a brief simulation study.

### 1.1. Contemporary Assumptions of Intelligence

In intelligence research, there is a broad consensus regarding the existence of general cognitive ability. Spearman (1904) observed that scores across diverse cognitive tasks tend to correlate positively, a phenomenon known as positive manifold, implying a common source of variance, typically denoted *g*. The Cattell-Horn-Carroll (CHC) theory of cognitive abilities (McGrew, 2009) provides a hierarchical framework for this structure: At the most abstract level resides *g*. *g* is composed of 16 broad abilities, such as fluid reasoning, comprehension-knowledge, and visual processing, that load on *g*. These broad abilities are further differentiated into numerous narrow abilities (e.g., induction as a narrow ability of fluid reasoning), operationalized by specific subtests or task variables (e.g., figural matrices to assess induction), which in turn load on their respective broad ability.

### 1.2. The Importance of Precise Intelligence Assessment

Intelligence is a core predictor across multiple life domains. It shows substantial associations with educational outcomes such as school grades (e.g., Roth et al., 2015), with job performance (e.g., Schmidt & Hunter, 2004), psychiatric disorders (e.g., Der et al., 2009), and life expectancy (e.g., Batty et al., 2009). Consequently, measures of intelligence are embedded in many diagnostic and decision-making frameworks. For example, intelligence tests are often used in educational and occupational selection. In legal contexts, specific intelligence thresholds inform determinations of legal competence and legal culpability: In Germany, individuals with intellectual disability are deemed not criminally responsible (Strafgesetzbuch [StGB], 1998, § 20), and in the United States, the death penalty cannot be applied to individuals with intellectual disability (Atkins v. Virginia, 2002; Hall v. Florida, 2014). Intellectual disability is commonly defined as an IQ below 70 (World Health Organization, 2019). In the upper range of the IQ scale, intellectual giftedness is most often defined as an IQ of 130 or higher (e.g., Baudson, 2025). Given the severity of such decisions, precise assessment of individual intelligence is essential.

### 1.3. Current Practices in IQ Assessment

In individual diagnostics, it is common to administer multiple cognitive tests to enhance the validity of the assessment. For example, when evaluating intellectual giftedness, typically at least two or three intelligence tests are conducted (e.g., Breuker et al., 2025; Steinheider, 2016). Composite scores based on multiple tests generally correlate more strongly with another intelligence measure than any single test alone (e.g., Brody, 2000). The rationale is that different intelligence tests place distinct content emphases (e.g., fluid vs. crystallized focus) and employ different types of stimuli (e.g., verbal, numeric, figural). A common method to combine results from multiple intelligence tests is simple arithmetic averaging. For instance, if an individual obtains IQs of 120, 125, and 130 on three different tests, the composite (unweighted) mean is 125.

However, simple averaging neglects two crucial aspects of combining scores intended to reflect the same construct: (1) Cognitive measures (and the tests assessing them) are typically positively correlated (cf. Spearman, 1904), but they are not statistically identical. As intercorrelations decrease, tests capture more non-overlapping variance, making it less likely that an individual will score uniformly high (or low) across all tests. An analogy may be drawn from a music talent show with three jurors: if their tastes are similar, their ratings will converge; if their tastes differ, receiving uniformly high ratings from all three jurors becomes more difficult. Consequently, a simple average tends to pull extreme profiles toward the middle, underestimating high ability and overestimating low ability. (2) When combining correlated scales, the variance of the composite is smaller than the variance of each test considered separately, with shrinkage increasing as intercorrelations decrease. In practice, neglecting this issue attenuates score extremes. Taken together, these considerations imply that, although convenient, simple averaging can be suboptimal and lead to serious misjudgment.

## 2. Exploratory Evidence on Test Score Combination Strategies

In Germany, eligibility to provide expert opinions on legal culpability requires only that the evaluator be a licensed psychologist (Bundesministerium der Justiz, 2023, No. 70(1)). Moreover, there are no statutory requirements for assessing intellectual giftedness. Against this background, we investigated which procedures psychologically educated individuals use to combine scores from multiple intelligence tests. To this end, we recruited a sample of *n* = 120 psychology students and *n* = 8 psychologists from Germany, Austria, and Switzerland (73.44% female, 23.44% male, 3.13% not reporting gender). Among the students, 34.17% were in their final bachelor’s semester and 45.83% in their final master’s semester (or an additional semester). Participants were presented with the following diagnostic case vignette: “Please imagine that you are an assessor at a center specializing in intellectual giftedness assessment. You are presented with a potential case to evaluate. After reading the case, provide your diagnostic judgment: Your client wishes to know whether they meet criteria for intellectual giftedness. You have them complete three intelligence tests with 100 items each. The three tests intercorrelate at *r* = 0.50 and, accordingly, load together on a general factor of intelligence (*g* factor). Each test has a reliability of *r* = 0.99. On one of the three tests, your client scores 1 standard deviation (SD) above the population mean; on another test, 2 SD above; and on the third test, 3 SD above.”

**Figure 1:**
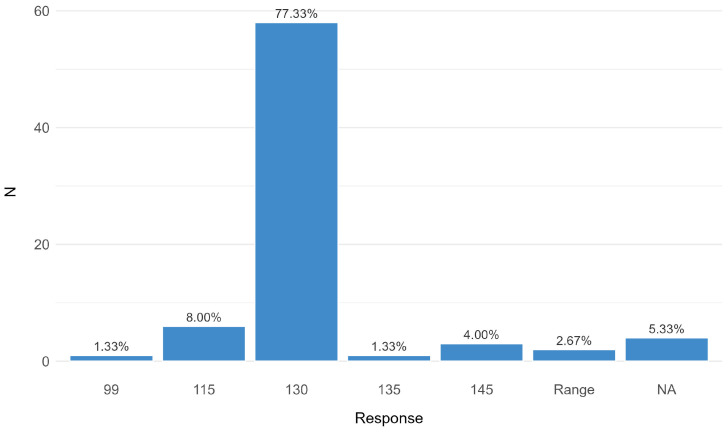
Distribution of responses in the IQ-combining task. Of the 75 participants, 71 provided an estimate, and 4 did not respond (NA). A total of 58 participants responded with the simple arithmetic average of the three IQ scores (130); only 1 participant (135) had a value close to the true IQ (137). Two participants reported a range (e.g., 115–145).

To ensure basic familiarity with the IQ metric, participants were first asked to report the mean and standard deviation of the IQ scale. Eighteen participants provided no response, 5 indicated they did not know, and 30 reported an incorrect mean or standard deviation. These 53 participants were excluded from subsequent analyses, leaving a final sample of *n* = 75. Figure 1 displays the distribution of IQ estimates for the case vignette among these participants. A total of 77.33% (*n* = 58) reported the simple arithmetic average of the three test scores (IQ = 130). Only one person reported a score (IQ = 135) close to the ‘true’ score of IQ = 137 (see Section 3.1), but without offering any explanation in a free-text field. Notably, no participant reported considering intercorrelations of the tests when forming their estimate. These findings highlight the need to promote valid procedures for aggregating intelligence test scores and to provide accessible tools for computing unified scores.

## 3. Formulas and Applications for Valid Unification

### 3.1. Formulas

To address the shortcomings of simple arithmetic averaging and to obtain a valid unified score *s_u_* from *t*-tests (*i* = 1, … *t*), we propose the application of the following formula:

(1) $$s_{u}=\frac{\sum_{i=1}^{t}b_{i}\times \left(s_{i}-M_{i}\right)}{\sqrt{Var_{s}+\;Var_{e}}}$$

where *b_i_* = weight assigned to the test *i* (typically 1 or a validity-based weight), *s_i_* = score on test *i*, *M_i_* = mean of test *i*. *Var_s_* is computed as

(2) $$Var_{s}=\overset{t}{\underset{i=1}{\sum }}b_{i}^{2}\times SD_{i}^{2}$$

and *Var_e_* as

(3) $$Var_{e}=\overset{t}{\underset{i=1}{\sum }}\overset{t}{\underset{j=i+1}{\sum }}2\times b_{i}\times b_{j}\times cor_{i,j}\times SD_{i}\times SD_{j}$$

where *SD_i_* = standard deviation of test *i* and *cor_i,j_* = intercorrelations of the tests.

Furthermore, the following formula serves to compute the reliability of unified tests:

(4) $$rel_{u}=\frac{\sum_{i=1}^{t}(b_{i}^{2}\times rel_{i})+T}{\sum_{i=1}^{t}(b_{i}^{2})+T}$$

where *rel_i_* = reliability of test *i* and

(5) $$T=\overset{t}{\underset{i=1}{\sum }}\overset{t}{\underset{j=i+1}{\sum }}2\times b_{i}\times b_{j}\times cor_{i,j}$$

**Figure 2:**
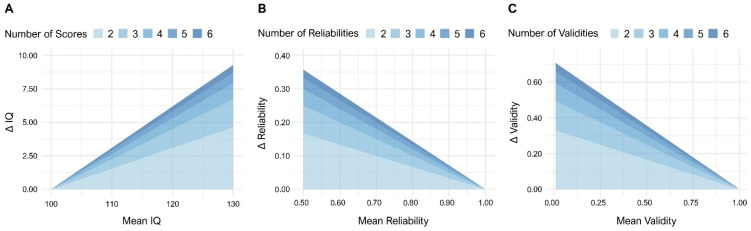
Differences between values computed with the formulas and the arithmetic averages. (**A**) illustrates the difference between the unified IQ computed with the formula and the arithmetic mean IQ, as a function of the arithmetic mean IQ and the number of IQ scores unified. With increasing mean IQ, simple averaging increasingly underestimates true IQ above the center of the IQ scale (100), and this underestimation grows logarithmically with increasing number of scores. (**B**) illustrates the difference for unified reliabilities. (**C**) illustrates the difference for unifying validities. For clarity, all panels assume a mean intercorrelation of *r* = 0.50 among the tests.

In addition, the unified external validity (i.e., the correlation of the unified tests with another variable) can be computed by:

(6) $$val_{u}=\frac{\sum_{i=1}^{t}b_{i}\times val_{i}}{\sqrt{\sum_{i=1}^{t}(b_{i}^{2})+T}}$$

where *val_i_* = validity of test *i*. Figure 2A–C illustrates the difference between the simple arithmetic averaging and the values derived from the proposed formulas.

These formulas are based on common and established variance rules for linear combinations and augment the method from Evans (1996) for the combination of two values to an arbitrary number of values.

### 3.2. R Package

Although the described formulas are conceptually straightforward and build on well-established principles of multivariate statistics and classical test theory, manually applying them each time scores must be unified is impractical. To facilitate this process, we developed an R package (version 4.5.2) named *unifyR* (version 1.0.0) (Weber et al., 2025a). The package provides three functions: *uniScore*, *uniRel*, and *uniVal*.

#### 3.2.1. uniScore

*uniScore* serves to unify the scores from multiple tests. The function is as follows:

*uniScore*(scores, M = NULL, SD = NULL, b = NULL, corm, method = “composite”) (7)

It requires users to provide the following parameters:

1. scores = a vector with a person’s scores from multiple tests;
2. M = a vector of means of the tests; must match the order of ‘scores’. Default: 0;
3. SD = a vector of standard deviations of the tests; must match the order of ‘scores’. Default: 1;
4. b = a vector of weightings for the test scores; must match the order of ‘score’. Default: 1;
5. corm = a matrix that contains the correlations between the tests. There must be ones on the diagonal;
6. method = the specification of the method for computing a unified score. “composite” (default) computes a non-latent unified score based on the Formulas (1)–(3). “pca” projects the standardized scores onto the first principal component of the intercorrelation matrix, which captures the linear combination of tests explaining the maximum shared variance. The principal component is derived from the population-level correlation matrix, and its loadings serve as weights applied to the individual’s scores.

The function returns the unified IQ score and additionally z, T, SW, and C scores, as well as percentile rank. Figure 2A shows an example of the function use.

#### 3.2.2. uniRel and uniVal

*uniRel* computes the reliabilities of a composite across multiple tests:

*uniRel*(rel, b = NULL, corm) (8)

The formula requires, apart from b and corm parameters, a vector rel of reliability coefficients of the tests.

*uniVal* computes the external validity (criterion correlation) of a composite across multiple tests:

*uniVal*(val, b = NULL, corm) (9)

**Figure 3:**
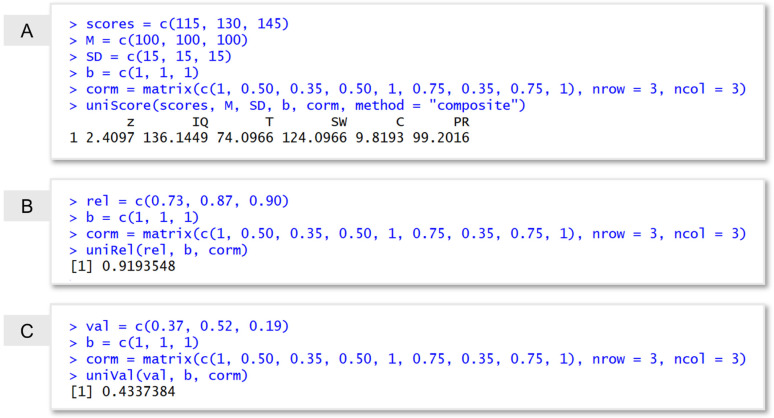
Examples of the functions from the *unifyR* package: (**A**) *uniScore*, (**B**) *uniRel*, (**C**) *uniVal*.

The formula requires, apart from b and corm parameters, a vector val of the validity of the tests. Figure 3B,C shows examples of the functions *uniRel* and *uniVal*.

### 3.3. Shiny Web Application

**Figure 4:**
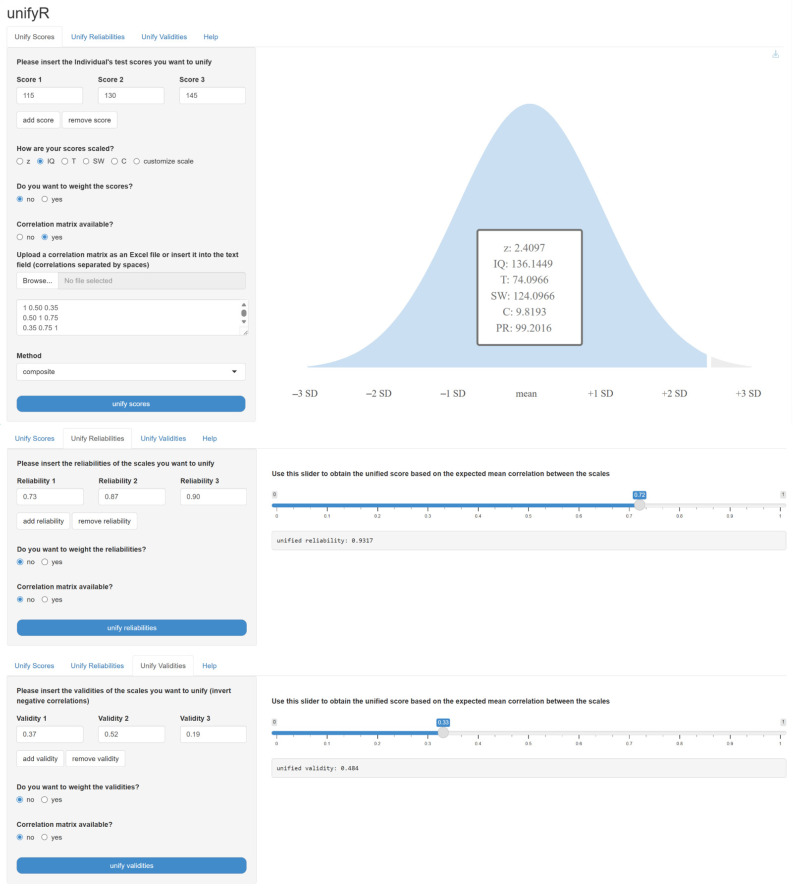
Shiny web application. The Shiny web application provides three functions analogous to the *unifyR* R package: unify scores, unify reliabilities, and unify validities. Users can enter the values from an arbitrary number of tests, optionally apply weights, and upload or input a correlation matrix. Since intercorrelations are not always available in practice, the application includes a slider to compute the unified value across different assumed mean intercorrelations.

To facilitate convenient application of the method without the need for a local R installation, we additionally developed an open-access Shiny web application. The application is available at: psychodiagnostics.shinyapps.io/unifyr (Weber et al., 2025b). Recognizing that intercorrelations among tests are not always known for a given test combination, the app includes an interactive sensitivity feature that computes the unified score across arbitrary correlation values. This functionality allows users to inspect the lower and upper bounds of the unified values as a function of assumed intercorrelations. Figure 4 provides a brief tutorial on using the web application.

## 4. Validation by Monte Carlo Simulations

To empirically validate the proposed method, we conducted Monte Carlo simulations, covering a range of plausible application scenarios. In each iteration, we generated a sample of *n* = 5000 participants, reflecting the typical magnitude of norm samples in intelligence testing. As validation criteria, we examined (a) the correlation between simulation results and the formula-based results, (b) mean bias, and (c) root mean square error (RMSE). In contrast to the simple averaging method, we also report absolute mean bias and RMSE between the simulated values and the average values. We used the R (R Core Team, 2026) packages *Matrix* (Bates et al., 2025), *matrixcalc* (Novomestky, 2022), and *simstudy* (Goldfeld & Wujciak-Jens, 2020) for data simulation, the packages *interactions* (Long, 2024) and *lm.beta* (Behrendt, 2023) for data analysis, and the package *ggplot2* (Wickham, 2016) for visualization. All R code and simulated datasets are available on OSF: https://osf.io/bafjs/overview?view_only=7e89ad4583e445279922e1298ab3fedb (accessed on 29 April 2026).

### 4.1. Unifying Scores

#### 4.1.1. Methods

We varied five simulation parameters to cover a broad range of scenarios: (1) The number *t* of test scores to be unified varied from 1 to 10. The mean correlation between the tests and the *SD* of the correlations were drawn randomly. Based on these parameters, *t* over 2 pairwise correlations between the *t*-tests were drawn. The actual (2) mean correlation and the actual (3) SD of the correlations were computed as further independent variables.

The target participant’s mean score was drawn randomly between 55 (mean of the IQ scale − 3 *SD*) and 145 (mean of the IQ scale + 3 *SD*). The *SD* of the participant’s scores was also drawn randomly between 0 and 30. Based on these two parameters, *t* random scores for the *t*-tests were drawn. From these scores, the actual (4) mean score and the actual (5) *SD* were computed as independent variables.

Participants’ test results were simulated on the *t* intelligence tests with the specified intercorrelations. For easier interpretability with respect to the IQ scale, we simulated participants who, on average, scored 100 points on each test with a *SD* of 15. We then imputed the randomly drawn scores of the target participant.

We next calculated each participant’s mean score across the *t*-tests. This mean was standardized and transformed to the IQ scale (IQ_simulated_). For the same scores and intercorrelations, the unified IQ was computed using the proposed formula (IQ_unified_). For each scenario (i.e., for each combination of the independent variables), we conducted 100 iterations and then computed the mean outcomes. In total, we performed 10,000 scenarios. For data analysis, we ran a multiple linear regression to determine whether the five independent variables could predict bias and conducted moderator analyses to examine whether the independent variables affected the correlation between the two IQ scores.

#### 4.1.2. Results

**Figure 5:**
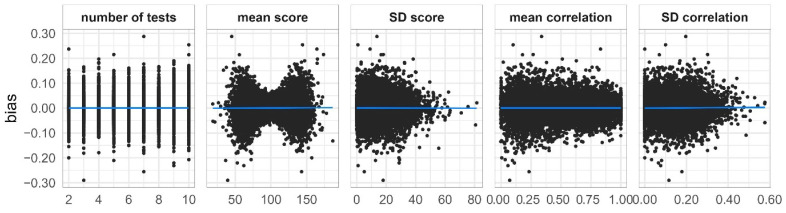
Correlation plots between independent variables and bias (unified scores). The figure shows bias between the unified IQ computed with the formula and the IQ resulting from the simulation for five independent variables. Bias ranged between ±0.30 IQ points. None of the independent variables predicted bias significantly (blue graphs).

The correlation between IQ_simulated_ and IQ_unified_ was *r* = 1.00, *p* < .001. Absolute mean bias was 0.04 IQ points, and RMSE was 0.04. In contrast, absolute mean bias between IQ_simulated_ and IQ_average_ of the simple average method was 8.66 IQ points, and RMSE was 12.92. None of the independent variables significantly affected bias between IQ_simulated_ and IQ_unified_, nor did they moderate the correlation between the two IQ estimates (Table 1). Figure 5 shows the prediction of bias by the independent variables. The variability of bias increases at more extreme IQ values (i.e., farther from the IQ scale mean). However, even in extreme IQ ranges, bias remains below 0.30 IQ points (i.e., less than 2% of the IQ scale *SD*).

### 4.2. Unifying Reliabilities

#### 4.2.1. Methods

We varied the following six simulation parameters: (1) the number of tests *t* to be unified, varied from 2 to 10, and (2) the number of items per test from 5 to 50. (3) The intercorrelations between the tests were drawn randomly between 0.00 and 1.00, and (4) the *SD* of the correlations was between 0.00 and 0.50. (5) The reliability of each test was drawn between *rel* = 0.50 and *rel* = 0.99, and (6) *SD* of the reliabilities between 0.00 and 1.00. We then combined the *t*-tests and computed the reliability of the combination (*rel_simulated_*). We also computed the unified reliability using the proposed formula (*rel_unified_*). We performed 10,000 scenarios. Finally, we conducted multiple linear regression and moderation analyses to determine the impact of the independent variables on bias and correlation.

#### 4.2.2. Results

**Figure 6:**
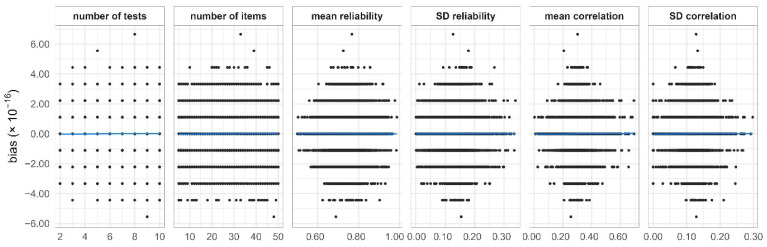
Correlation plots between independent variables and bias (unified reliabilities). The figure shows bias between the unified reliability computed with the formula and the reliability resulting from the simulation for six independent variables. Bias ranged between ±6.5 × 10^−16^. None of the independent variables predicted bias significantly (blue graphs).

The correlation between the simulated and unified reliabilities was *r* = 1.00, *p* < .001. Mean bias was −4.44 × 10^−19^, and RMSE 1.36 × 10^−16^. In contrast, the mean bias between simulated reliability and average reliability was 0.16, RMSE was 0.17. None of the independent variables impacted bias between the simulated and unified reliabilities significantly, nor did any moderate the correlation between the two reliability estimates (Table 2). Figure 6 shows the prediction of bias by the independent variables.

### 4.3. Unifying Validities

#### 4.3.1. Methods

We generated participants’ results on multiple correlated tests as described in Section 4.1, with correlations to an external criterion. We then calculated the empirical unified score and determined its correlation with the criterion (*val_simulated_*). We manipulated five independent variables: (1) the number of tests *t* from 2 to 10; (2) the mean validity varied from *val* = 0.00 to *val* = 1.00 as (3) the *SD* of the validities from 0.00 to 0.50; (4) the mean intercorrelations of the tests to be unified from 0.00 to 1.00, and (5) the *SD* of the correlations from 0.00 to 0.50. We performed 10,000 scenarios, and a multiple linear regression as well as moderation analyses to assess the impact of the independent variables on the outcomes.

#### 4.3.2. Results

**Figure 7:**
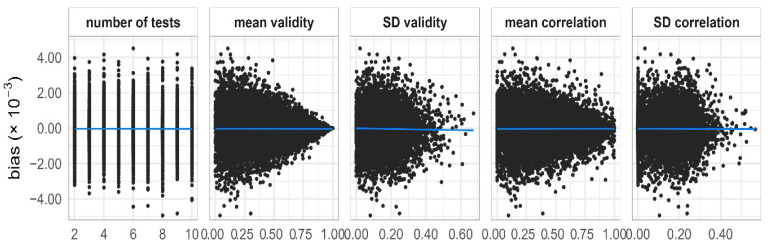
Correlation plots between independent variables and bias (unified validities). The figure shows bias between the unified validity computed with the formula and the validity resulting from the simulation for five independent variables. Bias ranged between ±5 × 10^−3^. None of the independent variables predicted bias significantly (blue graphs).

The correlation between the simulated and unified validities was *r* = 1.00, *p* < .001. Mean bias was −2.45 × 10^−5^, and RMSE was −8.03 × 10^−7^. In contrast, the mean bias between simulated and average validities was 0.12, and RMSE was 0.02. None of the independent variables significantly affected bias between simulated and unified validities (Table 3). The mean validity had a significant negative effect on the correlation between the simulated and unified validity, i.e., the correlation diminished with increasing test validity (*p* = .002). However, this effect was of negligible magnitude (β = −0.12 × 10^−4^). Figure 7 shows the prediction of bias by the independent variables.

## 5. Discussion

### 5.1. Fields of Application

The method and the accompanying applications were proposed as a variance-theoretically more consistent yet low-threshold alternative to the common simple arithmetic averaging approach. Although the R package also offers the possibility of a principal component-based integration, the method explicitly does not claim to replace regression-analytical, factor-analytical, or other latent modeling approaches. On the contrary, the method may be particularly suitable in situations where theoretical or practical considerations argue against more complex modeling. For instance, there are contexts in which the procedure used to integrate test scores must be as transparent and comprehensible as possible without requiring advanced statistical knowledge, particularly when external, governmental, or legal authorities request a diagnostic evaluation. Especially in highly sensitive selection procedures (e.g., student selection tests), a robust and communicable decision-making process can be preferred over an optimizing but model-dependent and politically less transparent approach. In such cases, the manifest method presented in this article may function as a valid alternative.

It should be noted that the formula for unifying test scores primarily affects the absolute level of the resulting composite rather than their relative ordering of individuals within a group. Hence, its practical relevance is greatest in contexts where diagnostic decisions depend on absolute scores (e.g., threshold-based classifications). Against the background that intelligence-related single-case diagnostics (e.g., the assessment of intellectual giftedness) typically involve the administration of multiple tests, we discussed the described method and the developed digital applications within the context of intelligence testing. Our empirical findings demonstrated that there is indeed a demand for low-threshold methods that allow multiple intelligence test results to be validly integrated. However, these findings should be interpreted considering the predominantly student-based sample. The results indicate that even psychologically educated individuals may default to simple averaging in the absence of explicit guidance. Nonetheless, to draw firm conclusions about applied practice, future research with larger and more professionally representative samples is needed.

Since the proposed method represents an application of established statistical and test-theoretical principles and does not claim to introduce a novel mathematical derivation, neither the method nor the corresponding digital tools are limited to the unification of intelligence tests. More generally, it may be used whenever multiple indicators need to be combined to support a diagnostic decision. However, a key prerequisite for such aggregation is construct comparability. In practice, tests may differ in content (e.g., reasoning vs. memory) or format (e.g., verbal vs. figural), and combining them without a clear conceptual rationale may lead to scores that are difficult to interpret. In the context of intelligence assessment, the CHC theory (McGrew, 2009) provides a widely accepted framework for structuring cognitive abilities. However, even comprehensive test batteries such as the WAIS (Wechsler, 2012) or the K-ABC (Kaufman & Kaufman, 2015) do not cover all broad CHC abilities. Depending on the diagnostic question, it may therefore be necessary to combine information across multiple tests. The *cross-battery assessment approach* (*XBA*; e.g., Flanagan et al., 2013) was developed to guide such decisions by supporting the theory-based selection of tests that meaningfully complement each other. In this sense, XBA addresses the substantive question of which measures can be combined, whereas the present method addresses the statistical question of how such measures can be combined once construct comparability has been established. While analytically distinct, these two levels of integration, the substantive and the statistical one, cannot be considered independently in practice: The method may also be applied in situations where latent modeling is not theoretically justified but where there is a practical need for an integrated decision criterion, since it is more comprehensible without advanced statistical knowledge. In such cases, however, the use of a composite score should be approached with caution, as it may obscure multidimensionality and reduce clarity regarding the substantive basis on which the diagnostic decision is made.

Finally, the method may further prove useful when multiple indicators of a test or an item are to be combined into an overall score. For example, the increasing digitalization of assessment has enabled access to process indicators (e.g., response time patterns or action sequences) that may provide insight into cognitive processes (cf. Stadler et al., 2023). If such process indicators are to be considered alongside classical test scores (e.g., number of correctly solved items) in diagnostic decision-making (perhaps because they enhance external validity), it is essential to integrate them appropriately while taking their intercorrelations into account.

### 5.2. Practical Considerations and Conditions of the Method

The proposed method is designed for situations in which intercorrelations between tests are either empirically available or can be reasonably approximated based on theoretical or conceptual considerations. However, in applied settings, the availability of such information may be limited, or available estimates may be imprecise due to small normative samples or limited comparability between tests. To address this issue, the accompanying Shiny app includes a sensitivity analysis feature that enables users to explore unified scores across a range of plausible intercorrelations, thereby supporting informed decision-making despite uncertain information about the tests. More generally, aggregation approaches can be understood as reflecting different levels of available information. When no information about intercorrelations is available, and no meaningful assumptions can be made, the aggregation problem reduces to estimating a central tendency based solely on observed test scores. In such cases, robust estimators (e.g., median-based approaches such as the Harrell-Davis quantile estimator; Harrell & Davis, 1982) may be preferable, as they provide increased stability in the presence of heterogeneous score profiles or potential outliers. At the same time, however, such approaches treat observed scores as interchangeable indicators and do not distinguish between shared and unique variance.

If, in addition to observed scores, information about the variability of single tests is available, this information can be incorporated through weighting schemes such as inverse-variance weighting. These approaches assign greater weight to more precise measures and may therefore represent a reasonable improvement over unweighted aggregation under limited information. However, they likewise do not account for intercorrelations between tests and thus ignore potential redundancy among measures. In the presence of positively correlated tests, this may lead to a bias toward the center of the scale. Taken together, these considerations suggest a hierarchy of aggregation strategies: as the amount of available structural information increases, more informed and theoretically consistent methods become applicable. While robust or variance-based approaches may be appropriate under conditions of limited information, the proposed method is preferable whenever intercorrelations can be estimated or meaningfully approximated.

### 5.3. Weighting

The proposed method serves to unify multiple tests while accounting for their intercorrelations and the corresponding variance shrinkage. Although the method and the accompanying digital tools allow for flexible specification of weights, the present article does not prescribe a single optimal weighting approach. Instead, the choice of weights depends on the available information and the intended application.

If no reliable information about differences in measurement precision or validity is available, equal weighting of the tests represents a reasonable default. However, if such information is available, alternative weighting strategies may be considered. For example, weights may be based on measurement precision, such as inverse-variance weighting, in cases where it is particularly important to ensure low uncertainty of the resulting unified score (e.g., in judgments of legal culpability). If the score is interpreted in relation to external criteria such as academic or job performance (e.g., in student selection tests), it may be preferable to use weights derived from external validities. It should be noted, however, that such weighting approaches do not account for intercorrelation and shared variance between the tests. For example, if two tests are both highly correlated with a criterion due to their shared variance, assigning them similar weights based on this criterion may effectively double-count this shared component.

To address this issue, shared variances between tests can be taken into account. Then it may be advisable to assign greater weight to tests that contribute more unique variance beyond the remaining measures, and less weight to those that exhibit redundancy due to high intercorrelations. This objective can be achieved by calculating weights as the product of the inverse intercorrelation matrix and an all-ones vector (i.e., *b* α *R*^−1^ 1).

In this sense, weighting decisions involve a trade-off between measurement precision, predictive validity, and the handling of shared variance. More generally, different weighting approaches reflect different optimization goals. For further insight, Chang (2009) and Wang and Stanley (1970) provide comprehensive overviews of methods for determining weightings.

## 6. Conclusions

The present article addressed a central issue in applied intelligence assessment, namely the widespread practice of computing arithmetic means from multiple intelligence tests. Our survey among psychologically educated individuals familiar with IQ metrics revealed that the vast majority relied on simple arithmetic averaging when asked to combine multiple intelligence test results. Although convenient, this procedure is statistically imprecise: averaging neglects that tests are imperfectly intercorrelated and that combining them reduces variance, leading to systematic overestimation of an individual’s intelligence below the IQ scale center and underestimation above the center.

To overcome these shortcomings, we proposed and validated the application of analytic methods for unifying test scores, reliabilities, and validities. Across Monte Carlo simulations, the analytic solution reproduced the empirical results with virtually perfect accuracy, independent of the number of tests and their intercorrelations. To bridge this methodology with practical usability, we introduced the R package *unifyR* and an accompanying Shiny web application as open-access tools to apply the method without advanced statistical training. These tools are directly applicable in diagnostic contexts such as educational placement or occupational selection, where decisions often hinge on small score differences.

## Figures and Tables

**Table 1:** Results of the multiple regression and the moderation analyses (unifying scores).

Variable	Bias				Moderation			
	*b* (×10^−4^)	β (×10^−4^)	*t*	*p*	*b* (×10^−4^)	β (×10^−4^)	*t*	*p*
NoT	0.53	31.16	0.30	.762	0.00	0.00	0.51	.612
*M_score_*	0.14	90.16	0.90	.368	0.00	0.00	1.00	.317
*SD_score_*	−0.23	−54.53	−0.54	.587	0.00	0.00	0.38	.698
*M_cor_*	3.65	21.46	0.21	.833	0.00	0.00	0.20	.843
*SD_cor_*	55.82	121.00	1.16	.247	0.00	0.00	0.30	.768

Note. NoT = number of tests, *M_score_* = mean of the scores, *SD_score_* = standard deviation of the scores, *M_cor_* = mean correlation between the tests, *SD_cor_* = standard deviation of the correlations between the tests. None of the independent variables predicted bias nor moderated significantly the correlation between the unified IQ computed with the formula and the IQ from the simulation.

**Table 2:** Results of the multiple regression and the moderation analyses (unifying reliabilities).

Variable	Bias				Moderation			
	*b* (×10^−4^)	β (×10^−4^)	*t*	*p*	*b* (×10^−4^)	β (×10^−4^)	*t*	*p*
NoT	0.00	54.10	0.48	.633	0.00	0.00	−1.64	.870
NoI	0.00	47.60	0.48	.634	0.00	0.00	−0.49	.626
*M_rel_*	0.00	−31.74	−0.28	.778	0.00	0.00	−0.05	.959
*SD_rel_*	0.00	0.69	0.01	.995	0.00	0.00	0.76	.445
*M_cor_*	0.00	57.72	0.52	.603	0.00	0.00	0.74	.458
*SD_cor_*	0.00	7.35	0.06	.949	0.00	0.00	0.26	.797

Note. NoT = number of tests, NoI = number of items per test, *M_rel_* = mean reliability, *SD_rel_* = standard deviation of the reliabilities, *M_cor_* = mean correlation between the tests, *SD_cor_* = standard deviation of the correlations between the tests. None of the independent variables predicted bias nor moderated significantly the correlation between the unified reliability computed with the formula and the reliability from the simulation.

**Table 3:** Results of the multiple regression and the moderation analyses (unifying validities).

Variable	Bias				Moderation			
	*b* (×10^−4^)	β (×10^−4^)	*t*	*p*	*b* (×10^−4^)	β (×10^−4^)	*t*	*p*
NoT	−0.02	−5.30	−0.51	.612	−0.11	−0.09	−1.22	.224
*M_val_*	−0.45	−12.60	−0.61	.545	−0.37	−0.12	−3.12	.002
*SD_val_*	−2.09	−21.90	−1.84	.065	−0.12	0.11	−1.06	.289
*M_cor_*	0.37	10.40	0.50	.619	−0.17	0.98	−1.77	.078
*SD_cor_*	0.84	8.50	0.69	.489	0.00	0.88	−0.02	.985

Note. NoT = number of tests, *M_val_* = mean validity, *SD_val_* = standard deviation of the validities, *M_cor_* = mean correlation between the tests, *SD_cor_* = standard deviation of the correlations between the tests. None of the independent variables predicted bias significantly. Only the mean of the validities moderated significantly the correlation between the unified validity computed with the formula and the validity from the simulation; however, this effect was of negligible magnitude.

## Data Availability

The original data presented in the study are openly available at https://osf.io/bafjs/overview?view_only=7e89ad4583e445279922e1298ab3fedb (accessed on 29 April 2026).
